# Early IgG Response to Foot and Mouth Disease Vaccine Formulated with a Vegetable Oil Adjuvant

**DOI:** 10.3390/vaccines7040143

**Published:** 2019-10-09

**Authors:** Xuemei Cui, Yong Wang, Babar Maqbool, Lijia Yuan, Shanshan He, Cenrong Zhang, Wei Xu, Songhua Hu

**Affiliations:** Department of Veterinary Medicine, College of Animal Sciences, Zhejiang University, 866 Yu Hang Tang Rd, Hangzhou, Zhejiang 310058, China

**Keywords:** adjuvant, soybean oil, vitamin E, ginseng saponins, foot-and-mouth disease

## Abstract

The present study evaluated soybean oil (SO) containing vitamin E (VE) and ginseng saponins (GS) (SO-VE-GS) for their adjuvant effect on foot-and-mouth disease (FMD) vaccine. Since mineral oil ISA 206 is a common adjuvant used in the FMD vaccine, it was used as a control adjuvant in this study. VE and GS were found to have a synergistic adjuvant effect. When mice were immunized with the FMD vaccine emulsified in SO with VE and GS, significantly higher serum IgG, IgG1, and IgG2a were found than VE and GS used alone. SO-VE-GS and ISA 206 behaved differently in adjuvant activities. When mice were immunized with the FMD vaccine adjuvanted with SO-VE-GS, significantly higher and earlier production of serum IgG was found than that adjuvanted with ISA 206. Although both adjuvants significantly increased the number of bone marrow plasma cells, a stimulation index of lymphocytes (SI) as well as the production of IL-4 and IL-6, SO-VE-GS promoted significantly higher SI and the ratio of CD4^+^/CD8^+^ T cells with production of increased IFN-γ and decreased TGF-β1 as compared with the ISA 206 group. The data suggested that SO-VE-GS activated Th1/Th2 immune responses. Transcriptome analysis of splenocytes showed that differentially expressed genes (DEGs), immune-related gene ontology (GO) terms, and Kyoto Encyclopedia of Genes and Genomes (KEGG) pathways were significantly enriched in the SO-VE-GS group. Therefore, the potent adjuvant effect of SO-VE-GS on the FMD vaccine may be attributed to the immune-related gene profile expressed in lymphocytes. Due to its plant origin and due to being much cheaper than imported mineral oil ISA 206, SO-VE-GS deserves further study in relation to vaccines used in food animals.

## 1. Introduction

Foot and mouth disease (FMD) is caused by the FMD virus (FMDV) and is a highly contagious disease of pigs, cattle, buffalo, sheep, and wild cloven-hoofed animals [1]. This disease spreads rapidly among susceptible animals and causes large economic losses [2]. FMD not only decreases animal’s meat and milk but is also the most important animal disease limiting the trade of animals and their products [3]. Therefore, the International Organization of Animal Health (OIE) listed FMD as a class A disease [4,5].

FMDV has a total of seven serotypes consisting of A, C, O, South African Territories (SAT) 1, SAT 2, SAT 3, and Asia 1 [6,7]. Serotype O is the most prevalent in China and some other Asian areas, and vaccination with inactivated vaccines is a routine approach to the control of FMD in many countries [8]. Since some types of immunity (e.g., Th1 cell vs Th2 cell, CD8^+^ vs CD4^+^ T cells, specific antibody isotypes) are not effectively achieved by the non-adjuvanted antigens, the adjuvant is often formulated in vaccines in order to improve the efficacy of vaccination [9,10]. The beneficial effects of formulation of adjuvants in vaccines can be found in various ways, including (a) increasing vaccine effectiveness to attain higher levels of immunogenicity and protective efficacy, (b) reducing the dose of antigen required for effectiveness, (c) increasing the speed of attainment of a protective immune response as well as reducing the number of immunizations required to achieve effectiveness, and (d) modulating the phenotype of T cell responses. In China, the commercial FMD vaccine adjuvant ISA 206 relies on foreign imports, whereas the available adjuvanted FMD vaccines have been reported to fail to elicit protective immune responses in some swine herds [11]. Consequently, this is a valuable source for a more suitable, effective, and economically relevant adjuvant, which improves the targeted immune responses to FMD vaccines.

Previous investigations showed that vegetable or mineral oil supplemented with saponins extracted from *Panax ginseng* C. A. Meyer exhibited adjuvant activity in FMD vaccines [12,13,14]. Vitamin E (VE) has been reported to exert immunostimulatory activities in various vaccines [15,16,17,18]. We hypothesized that a potent adjuvant effect on the FMD vaccine may be obtained when a vegetable oil is supplemented with GS in combination with VE. In the present study, a vegetable oil containing both GS and VE was evaluated for antigen effectiveness in FMD vaccination in mice. Since Montanide ISA 206 is the most widely used adjuvant in the FMD vaccine [19], this adjuvant was used as a positive control.

## 2. Materials and Methods

### 2.1. Animals

Since ICR (Institute of Cancer Research) mice are commonly used in immunological study, this strain was used in the present study. Female animals that were 6–8 weeks old were purchased from Shanghai Experimental Animal Center Co. Ltd. (Shanghai, China). Animals were kept at 24 +/− °C and 50% humidity in polypropylene cages with corncob bedding. Feed and water were supplied ad libitum.

### 2.2. Ethical Statement

All the experiments pertaining to animal use and their care strictly followed the Guidelines of Laboratory Animals of Zhejiang University and all the protocols were approved by Zhejiang University Animals Ethics Committee (ZJU20160377) on 4 March, 2016.

### 2.3. Adjuvants and Antigen

Soybean oil (SO) was obtained from Zhejiang Tian Yu Shan Medicinal Co. Ltd. (Zhejiang, China) and conformed to the standard of injection oil in the Chinese Pharmacopoeia. Standardized ginseng saponins (GS) was purchased from Hongjiu Ginseng Industry Co. Ltd. (Jilin, China), which contained ginsenosides Rb1 (18.9%), Rb2 (11.6%), Rc (10.2%), Rd (6.9%), Re (8.15%), Rg1 (3.5%), and Rf (1.58%), according to the analysis of high performance liquid chromatography (HPLC). Vitamin E (VE) was purchased from Sigma-Aldrich with purity ≥96% (Sigma-Aldrich, α-tocopherol, Cat. no. T3251, Saint Louis, USA). Montanite ISA 206 adjuvant was the product of Seppic Co. Ltd. (Shanghai, China). Inactivated FMDV type O antigen (strain O/Mya98/XJ/2010 + strain O/GX/09-7) was supplied by Tian Kang Biotech Co. Ltd. (Xinjiang, China) and the virus was inactivated by β-propiolactone. Different oil phases were made by dissolving VE and/or GS in DMSO so that each mL of the oil contains VE (100 μg) (SO-VE), GS (60 μg) (SO-GS) or both VE (100 μg) and GS (60 μg) (SO-VE-GS). Each ingredient dosed was based on our preliminary experiments (data not presented).

### 2.4. Preparation of FMD Vaccine

The inactivated FMDV type O antigen was diluted in physiological saline solution to a required concentration and then added to ISA 206 or SO in a 1:1 (*v/v*) ratio. The mixture was emulsified. The prepared vaccines contained 3 μg of 146 s per mL. The 146 s antigen of FMDV is essential to induce a protective immune response.

### 2.5. Immunization and Sample Collection

There are four experiments involved in this study.

**Experiment A.** To investigate the combined adjuvant effects of VE and GS on the FMD vaccine, mice were randomly assigned to five groups (*n* = 6/group) and were intramuscular (i.m.) immunized twice with 0.2 mL of FMDV antigen in saline solution or emulsified in SO, SO-VE-GS (VE 10 μg + GS 6 μg), SO-VE (10 μg), or SO-GS (6 μg) at a 2-week interval. Blood was collected 1 and 2 weeks after the booster immunization for detecting serum FMDV specific-IgG and IgG isotype.

**Experiment B.** To compare SO-VE-GS and ISA 206 for their stimulating effect on the production of antibody to FMD vaccine, mice were divided into three groups (*n* = 6/group) and were intramuscular (i.m.) immunized twice with 0.2 mL of FMDV antigen in saline solution or emulsified in SO-VE-GS or ISA 206 at a 2-week interval. Sera was collected 7 days before and 3, 7, 14, 21, 28, 35, and 42 days after the booster immunization to detect FMDV-specific IgG. Then the mice were euthanized to collect bone marrow cells for analysis of antibody-secreting bone marrow plasma cells (BMPCs).

**Experiment C.** To compare SO-VE-GS and ISA 206 for their effects on a cellular immune response, mice were divided into three groups (*n* = 6/group), and were intramuscular (i.m.) immunized twice with the FMDV antigen in saline solution or emulsified in SO-VE-GS or ISA 206 at a 2-week interval. Mice were sacrificed to aseptically collect spleens 2 weeks after the boost (the booster immunization). The cells were used for analysis of splenocyte proliferation, lymphocyte subsets, and cytokine production.

**Experiment D.** To investigate transcriptome profiling of gene expression in mice immunized with SO-VE-GS or ISA 206 adjuvanted vaccine, mice were divided into three groups (*n* = 4/group), and were intramuscular (i.m.) immunized twice with the FMDV antigen in saline solution or emulsified in SO-VE-GS or ISA 206 at a 2-week interval. Two weeks after the boost, mice were sacrificed to collect spleens for analysis of transcriptome sequencing.

### 2.6. Analysis of FMDV-Specific Antibody and Isotypes

Serum FMDV-specific IgG and IgG isotypes were measured by an indirect ELISA mainly as described by Zhang et al. [13]. Additionally, 96-well microtiter plates were loaded with 50 µL of rabbit anti-FMDV antibody (Lanzhou Veterinary Research Institute, Lanzhou, China) diluted in 0.05 M carbonate/bicarbonate buffer (pH 9.6, 1:10) per well. Then, it was incubated at 4 °C overnight. After washing with phosphate buffer saline containing 0.05% Tween-20 (PBST), the wells were blocked with 5% skimmed milk and incubated for 2 h at 37 °C. Plates were then washed with PBST, and 50 µL of the FMDV O antigen was added (1:10) with an incubation for 2 h at 4 °C. After that, 50 µL of serum (diluted 1:200 in PBS) was added and incubated for 1 h at 37 °C. Then, 50 µL of goat anti-mouse IgG (1:1000) or IgG1 (1:2000) and IgG2a (1:2000) (Santa Cruz Biotechnology, Inc., Santa Cruz, CA, USA) was added and incubated for 1 h at 37 °C. After washing, 50 µL of tetramethyl-benzidine (TMB) reagent was added to each well and incubated for 10 to 15 min at 37 °C. The reaction was terminated by adding 50 µL of 2 M H_2_SO_4_ Optical density (OD) at 450 nm, which was measured by a microplate reader (Thermo-Multiskan FC, Shanghai, China).

### 2.7. Lymphocyte Proliferation

The lymphocyte proliferation assay was conducted as described by Feng et al. [20]. The spleens were gently dissociated through a fine steel mesh into Hank’s balanced salt solution (HBSS, Sigma). The cell suspension was centrifuged at 1500 rpm for 5 min in 4 °C and the supernatant was discarded. After that, 3 mL of red blood cell lysis buffer (Beijing Solarbio Science and Technology Co., Ltd.) was added and cells were re-suspended for 3–5 min to lyse erythrocytes. After removing the buffer by centrifugation, splenocytes were re-suspended in complete medium (RPMI 1640 supplemented with 10% heat inactivated FCS). To a 96-well flat-bottom microtiter plate (Nunc), 100 µL of splenocytes (5 × 10^6^ cells/mL) were added. Then 100 µL of Con A (5 µg/mL), LPS (5 µg/mL), or the FMDV antigen (10 µg/mL) or medium was added, respectively. The wells with only medium were used as a control. The plates were first incubated for 44 h (37 °C, 5% CO2), and then the MTT method was used. The stimulation index (SI) was calculated based on the formula: SI = OD value from stimulated cells/OD value from unstimulated cells.

### 2.8. Cytokine Production by Splenocytes

To investigate the cytokine levels, 100 µL of splenocyte suspension (5 × 10^6^ cells/mL) and an equal volume of the FMDV antigen (10 µg/mL) were added into a 96-well flat-bottom microtiter plate (Nunc). The plate was incubated (37 °C, 5% CO2) for 72 h. After that, the supernatant was collected for determining cytokines by a commercial capture ELISA kit (Multi-Sciences Biotech Co., Ltd., Hangzhou, China). Concentrations of cytokines were calculated, according to the manufacturer’s instructions.

### 2.9. Flow Cytometry Analysis of T Lymphocyte Subsets

Splenocyte suspensions were adjusted to 5 × 10^6^ cells/mL. Following centrifugation at 1500 rpm for 8 min, the supernatant was removed. Splenocytes were stained with 1.5 µL of fluorochrome-conjugated anti-mouse antibodies to CD3e-APC (Clone: 145-2C11), CD4-FITC (Clone: RM4-5), and CD8a-PE (Clone: 53–6.7) (BD Biosciences, San Jose, CA, USA) for 30 min at room temperature. The cells were centrifuged (1500 rpm, 8 min) and then the supernatant was discarded. Cells were re-suspended in PBS before analysis by FACS LSR II flow cytometer (BD Biosciences). FLOW JO software (Treestar Inc., Ashland, OR) was used to analyze data.

### 2.10. Flow Cytometry Analysis of Bone Marrow Plasma Cells (Bmpcs)

Bone marrow cells were collected from mouse femur bones by flushing the marrow cavity with PBS [21,22]. Cells (2 × 10^6^ cells/mL) were centrifuged at 1500 rpm for 10 min and supernatants were removed. Anti-mouse CD16/CD32 (CD16 ‘Fc gamma III Receptor’ and CD32 ‘Fc gamma II Receptor’ are the low affinity receptors for the mouse IgG Fc portion and are expressed by B cells) antibody (1 µL) was added and incubated for 10 min on ice to block low-affinity Fc receptor expressed on other cells. Then cells were stained with 3 µL of fluorochrome-conjugated anti-mouse antibodies to CD38-Alexia (90/CD38, also known as Ab 90, CD38 is expressed at increasingly higher levels on B cells at each stage of B-cell differentiation, and is then down-regulated on germinal center B cells and mature plasma cells) and CD138-PE (Clone: 281–2, CD138 is expressed on B lymphocytes at specific stages during their differentiation). Then, the samples were incubated for 25–30 min at room temperature. After centrifugation (1500 rpm, 5 min), the cells were re-suspended in PBS and 2 µL of the 7-AAD reagent (all from BD Pharmingen) was used as a viability probe for dead cell exclusion before analysis by FACS LSR II flow cytometer (BD Biosciences). FLOW JO software (Treestar Inc., Ashland, OR) was used to analyze the data.

### 2.11. Transcriptome Sequencing

Splenocytes from mice immunized with the FMDV antigen in saline solution or emulsified in SO-VE-GS or ISA 206 were used for transcriptome sequencing. The total RNA of the spleen was extracted with RNAprep Pure Plant Kit (Tiangen Biotech (Beijing) Co., Ltd.). The quantity and quality of RNA were measured by Qubit 2.0 Fluorometer (Life Technologies, Carlsbad, CA, USA) and NanoPhotometer spectrophotometer (IMPLEN, CA, USA), respectively. All technical manipulations including transcriptome sequencing and assembly were carried out by Novogene Bioinformatics Technology Co., Ltd. (Beijing, China). Illumina sequencing libraries with 3 μg of RNA per sample were generated by the NEBNext UltraTM RNA Library Prep Kit (New England Biolabs, MA, USA,). Index-coded samples were clustered based on a cBot Cluster Generation System using TruSeq PE Cluster Kit v3cBot-HS (Illumia, CA, USA). After clustering, prepared libraries were sequenced on the Illumina Hiseq platform, and then the paired-end reads with 125 bp/150 base pairs were generated.

### 2.12. Analysis of Differentially Expressed Genes (DEGs)

The DESeq2 R package (1.16.1) was used for analyzing the differentially expressed genes. According to a negative binomial distribution model, DESeq2 can provide statistical routines to determine gene differential expression. Based on the Benjamini and Hochberg’s method, the resulting *P*-values were adjusted and used for detecting the false discovery rate, and adjusted *P* < 0.05 was deemed as differentially expressed genes in DESeq2. In addition, the GO and KEGG pathway enrichment analysis of the DEGs were based on the GO (http://www.geneontology.org/) and KEGG (http://www.genome.jp/kegg/) database, respectively [23].

### 2.13. Validation of Gene Expression by RT-qPCR

A total of 9 DEGs were selected to verify the RNA-seq results by RT-qPCR. The specific primers of reference [24] and primers sequences used for RT-qPCR (https://www.ncbi.nlm.nih.gov/) were synthesized by Sangon Biotech (seen in Table 1). Quantitative PCR was performed using SYBR^®^Premix Ex TaqTM II (Tli RNaseH Plus) on ABI7300 (PE Applied Biosystems, USA) and determined by the comparative CT method [25]. The PCR cycling conditions were followed as: pre-denaturation at 95 °C for 30 s, denaturation at 95 °C for 5 s, annealing and elongation at 60 °C for 31 s, and 40 cycles for each PCR program.

### 2.14. Statistical Analysis

SPSS 20.0 software (SPSS Inc., Chicago, IL, US) was adopted for data analysis. The data was analyzed by one-way ANOVA, which was followed by Duncan’s multiple test. Results were expressed as mean ± SE. *P* < 0.05, which was considered statistically significant.

## 3. Results

### 3.1. Synergistic Effect of Vitamin E and Ginseng Saponins on FMDV-Specific Antibody Response

**Figure 1:**
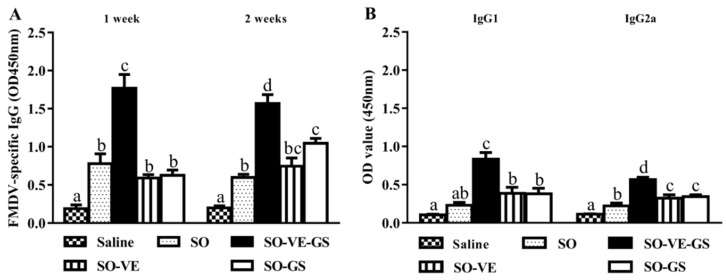
Serum FMDV(foot and mouth disease virus)-specific antibody response. Mice (*n* = 6/group) were i.m. immunized twice at a 2-week interval with the FMDV antigen in saline solution or emulsified in SO alone or SO with VE-GS (VE 10 μg + GS 6 μg) or VE (10 μg) or GS (6 μg). (**A**) Blood samples were collected 1 and 2 weeks after the boost to measure an FMDV-specific IgG. (**B**) IgG1 and IgG2a were measured 2 weeks after the boost. The values are presented as means ± SE. Bars with different letters are statistically different (*P* < 0.05).

To verify the combined effects of vitamin E (VE) and ginseng saponins (GS) on the FMD vaccine, mice were immunized with the FMD vaccine emulsified in soybean oil (SO) with or without VE and/or GS. Following immunization, blood was sampled for analysis of FMDV-specific IgG and IgG1 and IgG2a isotypes. As indicated, the serum IgG response induced by the FMD vaccine emulsified in SO was higher than the antigen in saline (Figure 1A). When compared with the vaccine emulsified in SO, the addition of GS clearly increased the IgG level at 2 weeks post boost. The highest IgG response was detected when the vaccine was emulsified in SO supplemented with both VE and GS. In the SO-VE-GS group, IgG is lower at 2 weeks than 1 week. Figure 1B shows that supplementation of SO with VE or GS enhanced IgG2a but not the IgG1 response when compared with the SO without a supplement. The highest IgG1 and IgG2a responses were elicited by the vaccine emulsified in SO supplemented with both VE and GS.

### 3.2. Effect of SO-VE-GS on the Duration and Early Production of Antibody Response

**Figure 2:**
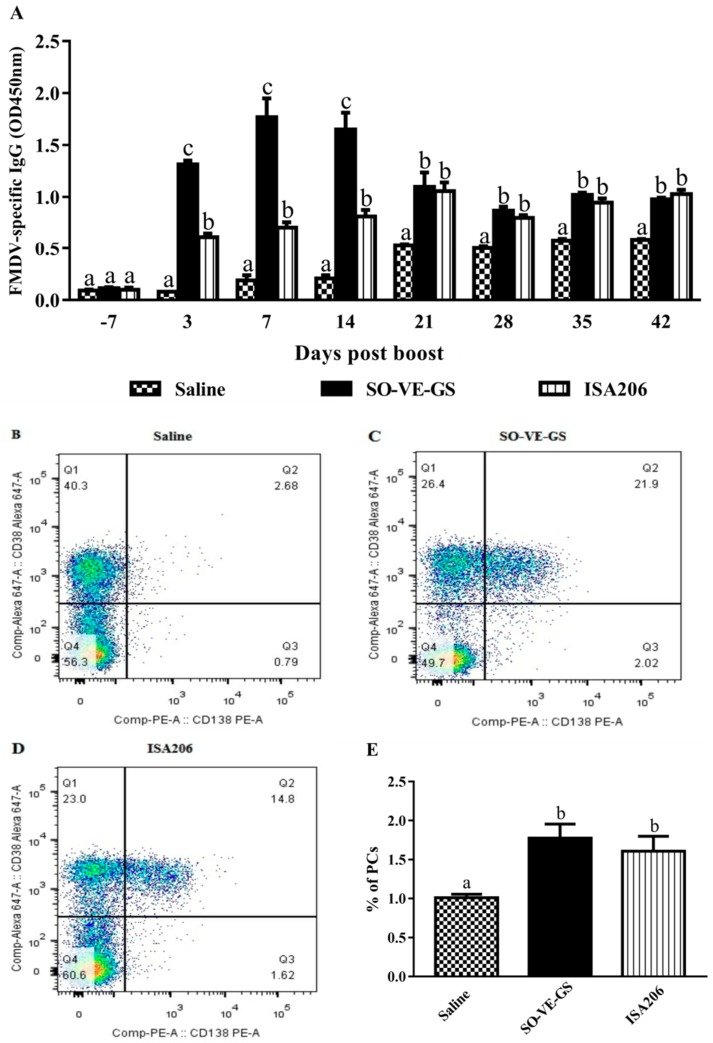
Timing and duration of serum FMDV-specific IgG responses and percentages of BMPCs in mice. Mice (*n* = 6/group) were i.m. immunized twice at a 2-week interval with the FMDV antigen in saline solution or adjuvanted with SO-VE-GS or ISA 206. (**A**) Blood samples were collected 7 days before and 3, 7, 14, 21, 28, 35, and 42 days after the boost to measure FMDV-specific IgG. (**B**–**D**) Bone marrow cells (**B**,**C**) were collected 42 days after the boost, and plasma cells (PCs) were identified as CD138^+^CD38^−^ cells by flow cytometry. (**E**) The percentages of PCs. The values are presented as means ± SE. Bars with different letters are statistically different (*P* < 0.05).

To evaluate SO-VE-GS for its effects on the production of serum FMDV-specific antibody response, mice were immunized with the FMD vaccine emulsified with SO-VE-GS or ISA 206. Mice immunized with the FMDV antigen in saline served as a control. Figure 2A shows that both SO-VE-GS and ISA 206 had an adjuvant effect on the FMD vaccine. After the first two weeks, the levels of the FMDV-specific IgG are higher with SO-VE-GS than with ISA206 (even as early as 3 days after the booster injection). However, from 21 days after the booster injection, there is no difference between the adjuvants. On analysis of BMPCs (CD138^+^CD38^−^), the adjuvant groups showed no significant difference in the number of PCs between them. However, both adjuvant groups had more PCs than the group with antigen in saline only (Figure 2B–E).

### 3.3. Effect of SO-VE-GS on Lymphocyte Proliferation

**Figure 3:**
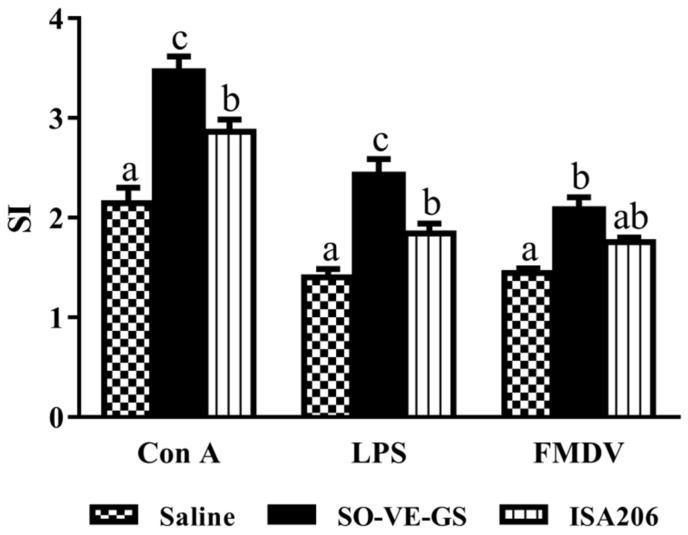
Lymphocyte proliferative responses. Mice (*n* = 6/group) were i.m. immunized twice at a 2-week interval with the FMDV antigen in saline or adjuvanted with SO-VE-GS or ISA206. Splenocytes were isolated from spleens harvested 2 weeks after the boost. The cells were cultured with Con A (5 µg/mL), LPS (5 µg/mL), or an inactivated FMDV antigen (10 µg/mL) for 48 h, respectively. The MTT method was used to measure cell proliferation, which was expressed as a stimulation index (SI). The values are presented as means ± SE. Bars with different letters are statistically different (*P* < 0.05).

To observe the effect of SO-VE-GS on lymphocyte proliferation, mice were immunized with the FMDV antigen in saline solution or emulsified with SO-VE-GS or ISA 206. Figure 3 shows lymphocyte proliferation in response to Con A, LPS, and the FMDV antigen, respectively. SI values from high to low were as follows: SO-VE-GS > ISA 206 > control (antigen in saline). SO-VE-GS gave a statistically higher lymphocyte stimulation index than ISA 206 when the cells were stimulated with Con A or LPS, but no significant difference between the two adjuvants when the cells were stimulated with the FMDV antigen.

### 3.4. Effects of SO-VE-GS on Cytokine Production by Lymphocytes

**Figure 4:**
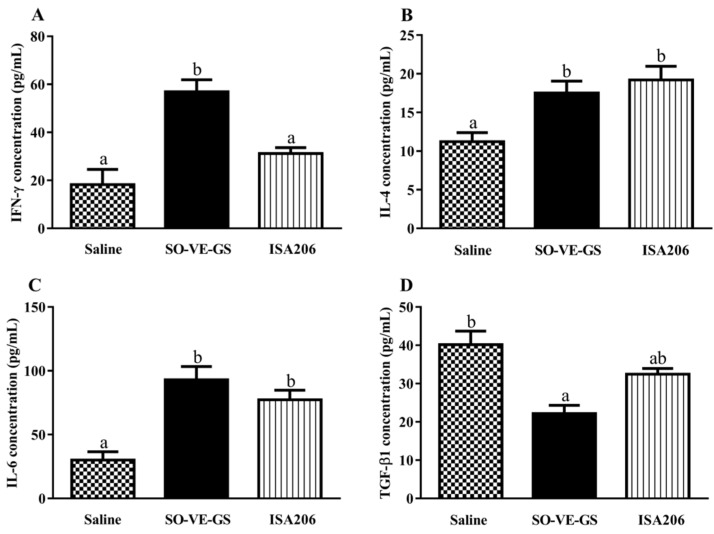
Cytokine production by splenocytes. Mice (*n* = 6/group) were i.m. immunized twice at a 2-week interval with the FMDV antigen in saline or adjuvanted with SO-VE-GS or ISA 206. Splenocytes were isolated from spleens harvested 2 weeks after the boost. The cells were co-cultured with an inactivated FMDV antigen (10 µg/mL) for 72 h. The supernatants were used for analysis of IFN-γ (**A**), IL-4 (**B**), IL-6 (**C**), and TGF-β1 (**D**) by ELISA kits. The values are presented as means ± SE. Bars with different letters are statistically different (*P* < 0.05).

Lymphocytes from mice in different groups were tested for their cytokine production when stimulated with the FMDV antigen. Figure 4 shows that significantly higher IFN-γ production was found only in lymphocytes from mice immunized with a vaccine adjuvanted with SO-VE-GS (Figure 4A), while lymphocytes from mice immunized with vaccines adjuvanted with SO-VE-GS or ISA 206 were detected to produce significantly higher IL-4 and IL-6 than the control (antigen in saline only). There was no difference in levels of IL-4, IL-6, and TGF-β1 production between the adjuvants themselves (Figure 4B–D). Regarding the production of TGF-β1 by lymphocytes, this cytokine was significantly lower in the SO-VE-GS group while only numerically lower in the ISA 206 group than the control (Figure 4D).

### 3.5. Effect of SO-VE-GS on CD4^+^/CD8^+^ T Cell Ratio

**Figure 5:**
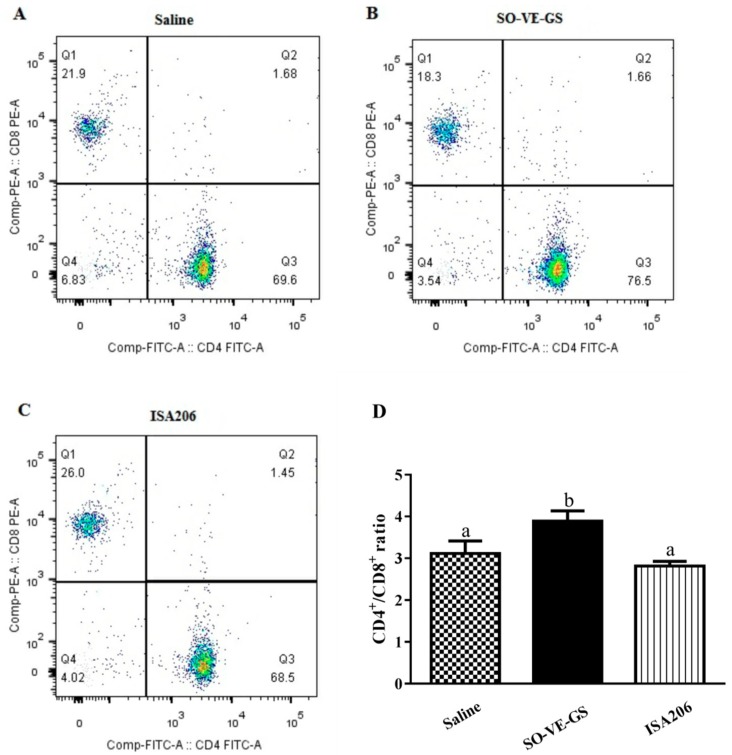
The CD4^+^/CD8^+^ T cell ratio of the splenocytes. Mice (*n* = 6/group) were i.m. immunized twice at a 2-week interval with the FMDV antigen in saline or adjuvanted with SO-VE-GS or ISA 206. Splenocytes were isolated from the spleens harvested 2 weeks after the boost. Cells were labeled with fluorochrome-conjugated antibodies to CD3-APC, CD4-FITC, and CD8-PE and analyzed by flow cytometry. The representative flow cytometry dot plots were shown in (**A**–**C**) and the quantification of CD4^+^/CD8^+^ ratio was shown in Figure (**D)**. The values are presented as means ± SE. Bars with different letters are statistically different (*P* < 0.05).

To observe the effect of SO-VE-GS on the ratio of CD4^+^/CD8^+^ T cells, splenocytes from mice immunized with a vaccine with different adjuvants were stained with CD4-FITC and CD8-PE and analyzed by flow cytometry. A significantly higher ratio of CD4^+^/CD8^+^ T cells was detected in T lymphocytes from the SO-VE-GS group while no significant difference was found between ISA 206 and the control (antigen in saline) (Figure 5).

### 3.6. Analysis of DEGs

**Figure 6:**
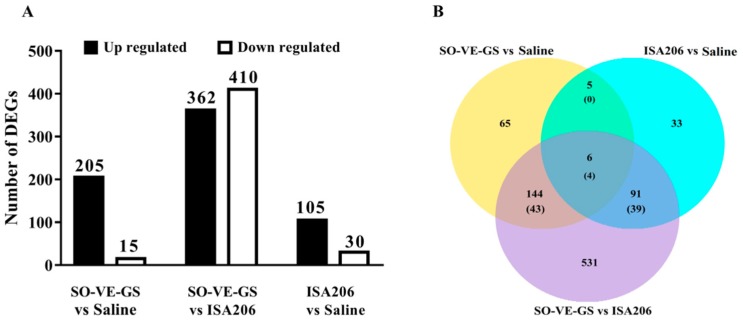
The numbers of differentially expressed genes (DEGs). Mice (*n* = 4/group) were i.m. immunized twice at a 2-week interval with FMDV antigen in saline or adjuvanted with SO-VE-GS or ISA206. Splenocytes were isolated from the spleens harvested 2 weeks after the boost and used for transcriptome sequencing. (**A**) The histogram shows the numbers of up-regulated and down-regulated genes in three groups. (**B**) Overlap comparison of differentially expressed genes enriched by GO analysis and the numbers of immune-related genes are in brackets.

**Figure 7:**
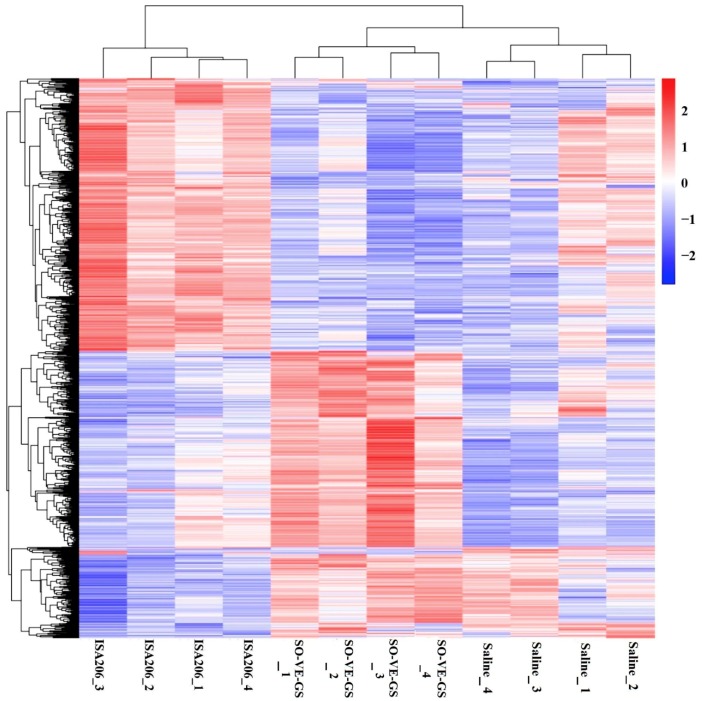
Heat map of hierarchical clustering of genes expressed in splenocytes from mice (*n* = 4/group) i.m. immunized twice at a 2-week interval with the FMDV antigen in saline or adjuvanted with SO-VE-GS or ISA206. The *P* values were adjusted using the Benjamini & Hochberg method. Corrected *P*-value of 0.05 and absolute fold change of two were set as the threshold for significant differential expression. The color key indicates reads per kilobase per million reads normalized log2 transformed counts. The red color represents high expression. The blue color represents low expression.

RNA-sequencing (RNA-seq) was used to analyze gene expression profiles of splenocytes from mice immunized with the FMDV antigen in saline solution or emulsified with SO-VE-GS or ISA 206. Compared with the antigen group, DEGs identified in SO-VE-GS and ISA 206 groups were 220 and 135, respectively. When compared with the ISA 206 group, the SO-VE-GS group had 772 DEGs identified with 362 genes up-regulated and 410 genes down-regulated (Figure 6A). Overlap genes that were significantly enriched in Venn are shown (Figure 6B). Six DEGs are shared in all groups, including four immune-related genes, including Cebpd, Camp, Cd177, and Fcnb. Camp was reported to be critical in maintaining colon microbiota balance and supports mucosal homeostasis, anti-inflammatory responses, and protection from carcinogenesis [26]. While mouse ficolin-B (Fcnb) was a functional member of the ficolin family activating complement via the lectin pathway [27]. In addition, 5, 91, and 144 DEGs were commonly shared in every two comparison groups with immune-related genes of 0, 39, and 43, respectively. The immune-related genes were searched based on the Mouse Genome Informatics (MGI) database (http://www.informatics.jax.org/vocab/gene_ontology/GO:0002764). The hierarchical heat map demonstrated the visual summary of the differences between the transcriptional responses to different groups. Figure 7 shows that different visuals of hierarchical clustering of genes were expressed in the splenocytes when animals were immunized with the FMDV antigen with different adjuvants. Yet, clusters of the two adjuvant groups were similar when compared with the saline group (antigen in saline only). DEGs identified in biological replicates clustered together, which indicates good reproducibility of treatments.

### 3.7. Gene Ontology Enrichment Analysis of DEGs

**Figure 8:**
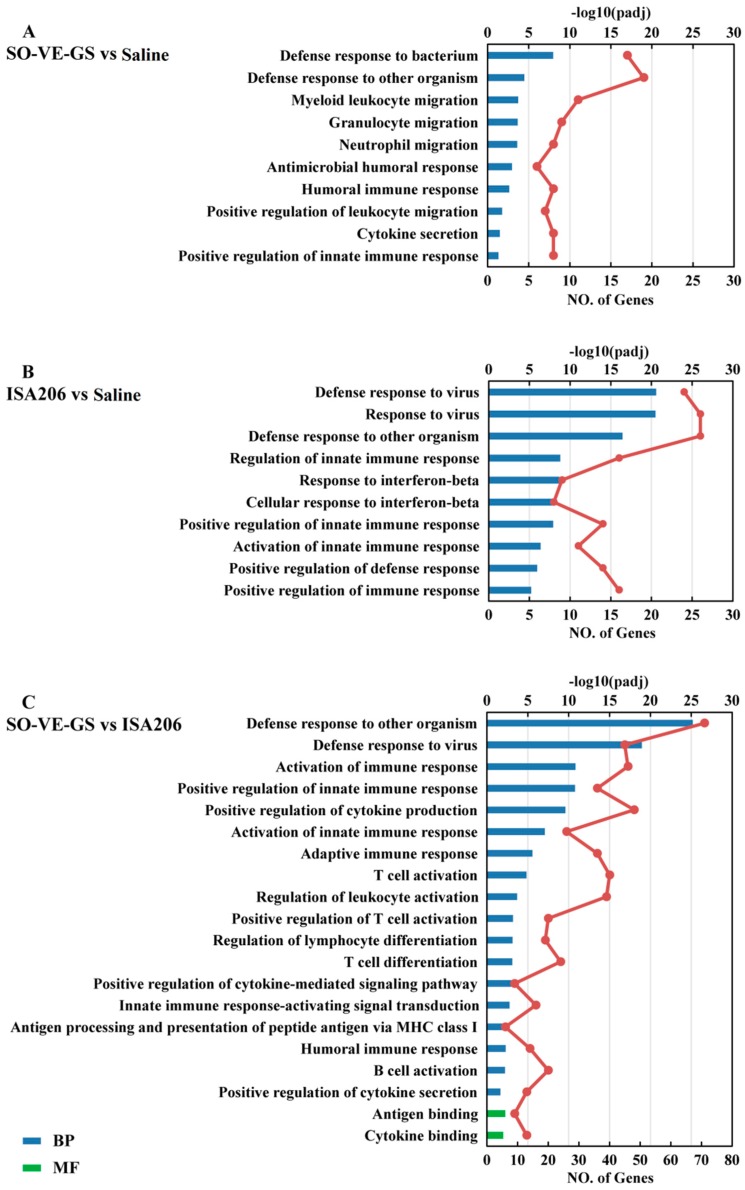
GO functional enrichment analysis. The GO terms related to immune responses were classified based on Gene Ontology. DEGs of (**A**) SO-VE-GS vs Saline, (**B**) ISA206 vs Saline, and (**C**) SO-VE-GS vs ISA206 were used for GO functional enrichment analysis. GO terms with a corrected *P* value less than 0.05 were considered significantly enriched by differentially expressed genes.

In order to functionally analyze the obtained genes, DAVID (Data-base for Annotation, Visualization, and Integrated Discovery) [28] was performed for GO (Gene Ontology) [29] enrichment analysis. GO-terms were classified into three categories including the Biological Process (BP), Molecular Function (MF), and Cellular Component (CC). We performed enrichment analysis on the top 10 to 20 immune-related GO terms mainly assigned to the BP category. Only two in SO-VE-GS vs ISA 206 fall into the MF category. The SO-VE-GS group was identified to have significantly activated GO terms related to the defense response to bacteria, the defense response to other organisms, immune cell migration, the humoral immune response, and cytokine secretion when compared with saline (Figure 8A) and significantly higher expressed genes related to various immunological responses including activation of the innate immune response, the adaptive immune response, the humoral immune response, T/B cell activation, antigen processing and presentation, positive regulation of cytokine production, and antigen/cytokine binding (MF) when compared with the ISA 206 group (Figure 8C). Meanwhile, the GO terms of ISA 206 vs saline had a high concordance with the SO-VE-GS vs ISA 206, especially in relation to the regulation of the innate immune response, like the defense response to the virus, the defense response to other organisms, regulation of the innate response, positive regulation of the innate response, and activation of the innate response (Figure 8B).

### 3.8. Kyoto Encyclopedia of Genes and Genomes Pathway Enrichment Analysis of DEGs

**Figure 9:**
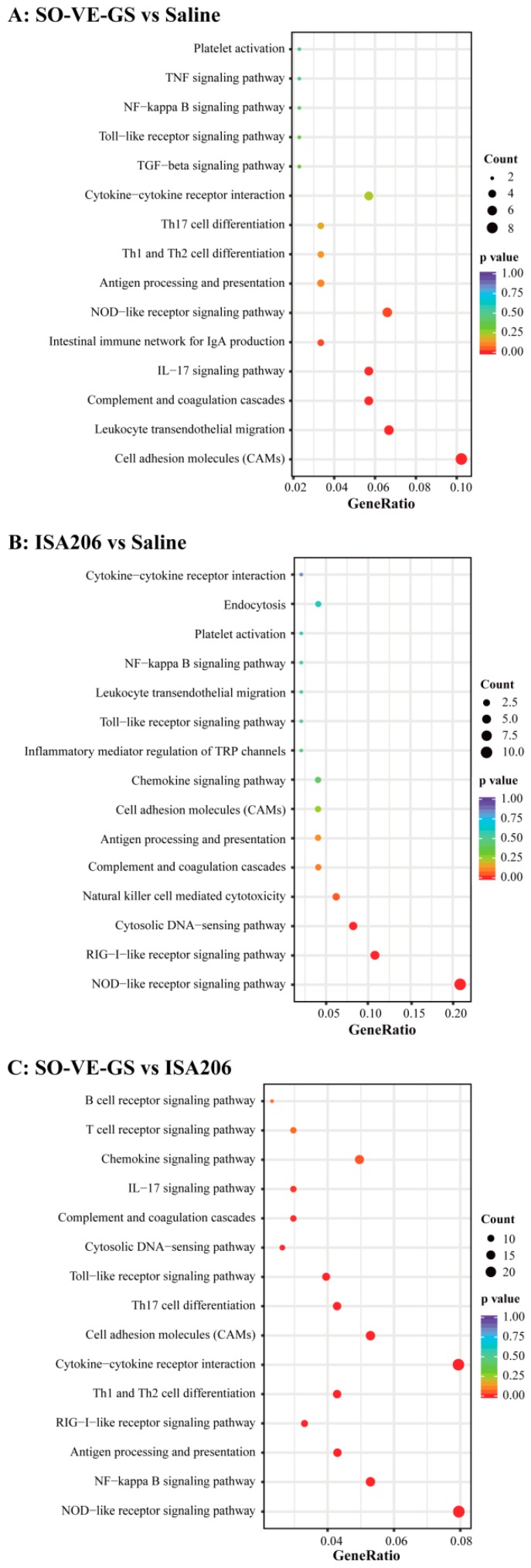
Enrichment of KEGG pathways analysis. DEGs of (**A**) SO-VE-GS vs Saline, (**B**) ISA206 vs Saline, and (**C**) SO-VE-GS vs ISA206 were used for KEGG pathway analysis. The X-axis represents the value of the gene ration. The Y-axis represents the enrichment term of the pathway. The size of the dot represents the number of DEGs. The color represents an adjusted *P* value.

KEGG (Kyoto Encyclopedia of Genes and Genomes) is a database resource containing a collection of pathway maps on molecular interaction and reaction networks [30]. In this study, 15 immune-related pathways were selected for analysis of KEGG pathways. Figure 9A implies that, compared to saline (antigen in saline), six of 15 immune related pathways were significantly activated by SO-VE-GS, and they were the cell adhesion molecules (CAMs), leukocyte transendothelial migration, complement and coagulation cascades, IL-17 signaling pathway, intestinal immune network for IgA production, and the NOD-like receptor signaling pathway. In addition, four pathways (Th1 and Th2 cell differentiations, TGF-beta signaling pathway, and TNF signaling pathway) were specific to SO-VE-GS. Compared with ISA 206, 13 of 15 immune related pathways were significantly activated in the SO-VE-GS group (Figure 9C). In the SO-VE-GS group, pattern recognition receptors (PRRs) signaling pathways (NOD-like receptor signaling pathway, RIG-I-like receptor signaling pathway, Toll-like receptor signaling pathway, and the downstream NF-kappa B signaling pathway) and immune-related pathways (cytokine-cytokine receptor interaction, complement and coagulation cascades, Th1 and Th2 cell differentiation, and antigen processing and presentation) were predominant, especially the NOD-like receptor signaling pathway and cytokine-cytokine receptor interaction. In the ISA 206 vs saline comparison, four of 15 immune related pathways were significantly activated. These were the NOD-like receptor signaling pathway, the RIG-I-like receptor signaling pathway, the cytosolic DNA-sensing pathway, and natural killer cell mediated cytotoxicity. The exception is that ISA 206 activated three pathways that were clearly different from the other two groups, with natural killer cell mediated cytotoxicity, inflammatory mediator regulation of TRP channels, and endocytosis (Figure 9B).

### 3.9. Validation of Gene Expression by Real Time-qPCR

**Figure 10:**
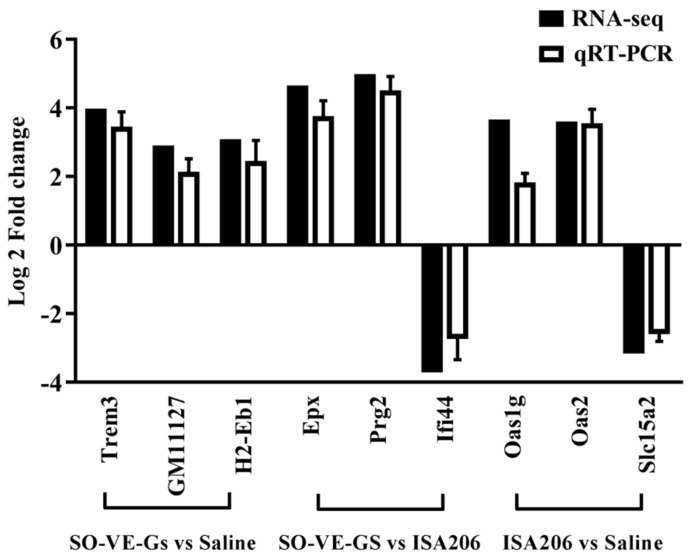
Validation of DEGs by RT-qPCR. Data were normalized to the expression of GAPDH, and the log two-fold changes were calculated in comparison to the corresponding groups.

To validate the results from RNA-seq, nine DEG candidates were randomly selected that were up-regulated or down-regulated and measured gene expression by RT-qPCR. Data from RT-qPCR were highly correlated with those from RNA-seq (Figure 10), which suggests the reliability of RNA-sequence analysis.

## 4. Discussion

We have previously demonstrated the combined adjuvant effects of mineral oil or vegetable oil supplemented by ginseng saponins (GS) [11,12,13,14]. The present study showed that injection of an FMD vaccine emulsified in SO containing GS and VE (SO-VE-GS) in mice induced significantly higher IgG, IgG1, and IgG2a than those containing GS or VE alone. Supplement of saponins in oil emulsion to increase adjuvant activity has been reported previously. Xiao et al. reported that injection of an oil-emulsified FMDV antigen added with Quil A in pigs induced significantly increased serum hemagglutination titers when compared with the vaccine without a supplement [31]. Song et al. found that GS and a mineral oil worked together to promote the immune response to the FMD vaccine in a mouse model [12]. Zhang et al. reported that injection of the FMDV antigen emulsified with vegetable oils such as RO and SO containing GS induced significantly higher Th1/Th2 immune responses in mice when compared with RO, SO, or GS used alone [13,14]. Similarly, in the present study, supplementation of GS in SO has been found to increase antibody responses 2 weeks after the booster immunization. VE has been reported to exert immunostimulatory activities as an adjuvant to various vaccines and formulated in AS03 [15,16,17,18]. The present study found similar results that addition of VE in SO increased IgG1 numerically and IgG2a significantly. We found that VE in combination with GS in SO significantly increased IgG, IgG1, and IgG2a when compared with VE or GS used in SO alone (Figure 1). The increased antibody responses in the SO-VE-GS group may be attributed to the orchestrated adjuvant activities of SO, VE, and GS.

Specific humoral responses are essential for protecting susceptible animals against infection by FMD [32,33]. Serum antibody levels in animal are thought to have a direct relation to the protective effect against FMDV infection [1]. Our results showed that SO-VE-GS and ISA 206 exhibited adjuvant activity and promoted significantly higher serum IgG levels than the antigen in saline (Figure 2A). However, the IgG responses fluctuated differently in the two groups. In the SO-VE-GS group, the antibody response grew rapidly to the peak seven days after the booster immunization. However, in the ISA 206 group, the IgG level rose progressively to the peak at 21 days after the booster immunization. Prior to 21 days after the booster immunization, the IgG level was significantly higher in the SO-VE-GS group than in the ISA 206 group, which indicates that the SO-VE-GS stimulated early production of the antibody response as compared with the conventional ISA 206. During the period of 21 to 42 days after the booster immunization, no statistical difference was found in serum IgG levels in both adjuvant groups. The discrepancy between two groups is related to the characteristics of SO-VE-GS and ISA 206 but the detailed mechanism is unclear. Plasma cells are differentiated from activated B lymphocytes, and the main function is to secrete antigen-specific antibodies [34]. The cells that home to and persist in the bone marrow continue secreting antigen-specific antibodies and act as important cells in the long-term protection responses against pathogenic agents [35,36]. The long-lasting IgG response was associated with the enhanced FMDV-specific antibody-secreting BMPCs with the adjuvants (Figure 2B–E). In our study, significantly more FMDV-specific antibody-secreting plasma cells were detected in adjuvant groups, which suggests that both adjuvants stimulated the production of long-lived IgG bone marrow plasma cells.

Different subtypes of IgG, such as IgG1 and IgG2a, provide animals with the bulk of immunity against the majority of infectious agents. During T cell-dependent immune responses, the major IgG subtypes involved in the development of the immune procedure progressively change [37]. T lymphocytes and their secreted cytokines are responsible for these changes. In mice, IL-4 and IL-6 are preferred to switch activated B cells to produce the IgG1 antibody (Th2) [38,39,40,41] and IFN-γ is associated with boosting the IgG2a response (Th1) [42], while regulatory T (Treg) cells usually regulate the function of Th1/Th2 by employing cytokine TGF-β1 [43]. The results showed that SO-VE-GS promoted significantly higher IgG1 and IgG2a (Figure 1B). The increase of IgG1 and IgG2a is also related with increased IL-4 and IFN-γ (Figure 4) and indicated that SO-VE-GS activated both Th1 and Th2 responses. TGF-β1 can originate from T and B cells and is believed to be a cytokine capable of inhibiting production of IFN-γ [44]. Significantly increased production of IFN-γ can be explained by significantly decreased TGF-β1 in the SO-VE-GS group while no significant changes have been found for these cytokines in the ISA 206 group (Figure 4). Lymphocyte proliferation varies depending upon the different stimulating mitogens used. To induce largely effective antibody production, activated B lymphocytes require extensive clonal expansion [45]. In the SO-VE-GS group, significantly increased splenocyte proliferation was found after ConA, LPS, or FMDV antigen stimulation (Figure 3), which implies that both T and B cells were provoked. The result of the improved lymphocyte response is in agreement with the increased serum IgG levels observed in mice, which were injected with the FMDV antigen adjuvanted with SO-VE-GS.

CD4^+^ and CD8^+^ T cells are the major subsets of T lymphocytes. CD4^+^ T cells mainly promote B cell maturation and facilitate the antigen presentation of APCs, while CD8^+^ T cells are associated with killing the target cells [46]. The CD4^+^/CD8^+^ ratio is an old biomarker for HIV and a low CD4^+^/CD8^+^ ratio predicts immunosenescence in HIV-infected adults with increased morbidity [47,48]. In pigs persistently infected with classical swine fever viruses (CSFV), increased CD8^+^ T subset and decreased CD4^+^ T subset have been found [49]. The CD4^+^ T cell response plays an important role in generating the optimum antibody responses to the FMD vaccine in cattle [50]. It was reported that the ratio of CD4^+^/CD8^+^ T lymphocytes was increased when mice were inoculated with OVA in combination with polysaccharides from *Agaricus blazei* Murrill [51]. In this study, a significantly higher ratio of CD4^+^/CD8^+^ T cells was detected in the SO-VE-GS group as compared with either the control (antigen in saline) or the ISA 206 group, which indicates that more CD4^+^ T cells were activated in the SO-VE-GS group.

SO-VE-GS and ISA 206 exhibit different behaviors in their adjuvant effects on the immune response to the FMDV antigen. This dissimilarity may be caused by different genes expressed by lymphocytes in the two groups. RNA-sequence based transcriptome analysis is a tool that allows a deep understanding of the complicated physiological pathways, including the immune responses [52,53,54,55]. However, only a few reports have been found on the use of the transcriptome to analyze the effects of vaccine adjuvants. Recently, transcriptome analysis was used to study the role of adjuvants in rabies vaccine immunization and found that there were significant differences in gene expression between the mice immunized with different type of adjuvants, except for two aluminum adjuvant vaccines, which induced similar gene expression [56]. In the present study, more significantly DEGs were found in the SO-VE-GS group than in the ISA 206 group by RNA-sequence analysis (Figure 6A). Although both SO-VE-GS and ISA 206 were used as an oil phase to emulsify the FMD vaccine in the present study, GO analysis shows that only four of the immune-related DEGs were commonly shared by the two groups (Figure 6B). Clustering analysis, which is a study of setting the genes with the same or similar expression patterns together [57], showed that two groups had apparently different patterns of hierarchical clustering of genes expressed in splenocytes (Figure 7).

Innate and adaptive immunity operate in cooperative and interdependent ways. The innate immune system delivers signals that are essential to induce and direct subsequent adaptive immunity [58]. When protein antigens are introduced in the body, they are engulfed by the resident antigen presenting cells (APC) and degraded via the processing pathway, which results in peptides loaded onto MHC molecules [59]. These complexes are then transported to the surface of the cells. T lymphocytes are activated by recognition via TCR of an antigen peptide-MHC complex on APC [60]. After activation, these T helper cells produce various kinds of cytokines, which play a very central role in the activation of B cells and other cells that participate in the immune response. In this study, GO enrichment analysis showed that the SO-VE-GS group has significantly activated the GO terms that related to various immunological responses including activation of the innate immune response, the adaptive immune response, the humoral immune response, T/B cell activation, antigen processing and presentation, positive regulation of cytokine production, and antigen/cytokine binding (MF) when compared with the ISA 206 group (Figure 8C). KEGG analysis showed that SO-VE-GS group significantly improved the activation of immune-related pathways than the ISA 206 group (Figure 9C). Two predominant signal pathways in the SO-VE-GS group were found: pattern recognition receptors (PRRs) signaling pathways (like NOD-like receptor signaling pathway) and the immune-related pathways (like cytokine-cytokine receptor interaction). These results indicated that the adjuvant of SO-VE-GS can strongly stimulate the immune system in mice.

## 5. Conclusions

In summary, VE and GS exert a synergistic adjuvant effect on the immune responses to the FMD vaccine emulsified in SO. SO-VE-GS and ISA 206 behave differently in their adjuvant activity. When mice were immunized with the FMD vaccine emulsified in SO containing both VE and GS (SO-VE-GS), significantly higher and earlier production of serum FMDV-specific IgG was found than in the ISA 206 group. Although both adjuvants significantly increased the number of BMPCs, an enhanced lymphocyte proliferation response promoted the production of IL-4 and IL-6. SO-VE-GS promoted a significantly higher lymphocyte proliferative response and the CD4^+^/CD8^+^ T cell ratio with production of increased IFN-γ and decreased TGF-β1 by lymphocytes, as compared with the ISA 206 group. These data suggested that SO-VE-GS activated Th1/Th2 immune responses. Transcriptome analysis of splenocytes showed that DEGs, immune-related GO terms, and KEGG pathways were significantly enriched in the SO-VE-GS group. Therefore, the potent adjuvant effect of SO-VE-GS on the FMD vaccine may be attributed to the immune-related gene profile expressed in lymphocytes. Due to its plant origin and being much cheaper than imported mineral oil ISA 206, SO-VE-GS deserves further study in relation to vaccines used in food animals.

## Figures and Tables

**Table 1:** Sequences of primers for quantitative RT-PCR.

Gene	Primer Sequence
*(a) GAPDH*	Forward: 5-TCGTCCGGTAGACAA AATGG-3 Reverse: 5-GAG GTC AATGAAGGGGTCGT-3
*(b) Trem3*	Forward: 5-CTGATGCTCTGTGTCTCGGG-3 Reverse: 5-AGGACAAGTCAGGGTCAGGT-3
*(c) Gm11127*	Forward: 5-GCCTCTTCCATCCACCGACT-3 Reverse: 5-TCATCATAAAAGCCACCACAGC-3
*(d) H2-Eb1*	Forward: 5-ATAAATTCCTTGTGCGGCGG-3 Reverse: 5-CAGGAGGTTGTGGTGTTCCA-3
*(e) Epx*	Forward: 5-TTAGCCACACTCATCCTCACC-3 Reverse: 5-TGCTATGCAGTCTCGAAGGAC-3
*(f) Prg2*	Forward: 5-TCGCCTCCATCCACAGTTTC-3 Reverse: 5-CCATCAACCCATCGAAAGCG-3
*(g) Ifi44*	Forward: 5-ATGGCATTCTGCATTTGGCT-3 Reverse: 5-AATGCCTCCAGCTTGGACTT-3
*(h) Oas1g*	Forward: 5-AGCCTAATCCCTTAATCTACACC-3 Reverse: 5-GCAGGTAGAGAACTCGCCAT-3
*(i) Oas2*	Forward: 5-CCTTGGAAAGTGCCAGTACCT-3 Reverse: 5-TGCCACAAGATCCCTCCTGTA-3
*(j) Slc15a2*	Forward: 5-GCAGAGGCACGGACTAGATAC-3 Reverse: 5-GGGATCAACGGCTGTTCACAT-3
